# Facebook Enhances Antidepressant Pharmacotherapy Effects

**DOI:** 10.1155/2014/892048

**Published:** 2014-01-19

**Authors:** Jorge Mota Pereira

**Affiliations:** Clínica Médico-Psiquiátrica da Ordem, Rua Gonçalo Cristóvão, No. 347, Sala 202, 4000-270 Porto, Portugal

## Abstract

Treatment-resistant major depressive disorder (TR-MDD) is a complex condition, with very low remission rates. In recent years some studies have been conducted on the implementation of cognitive behavioral therapy and psychodynamic psychotherapy interventions via the Internet to MDD patients, and results have been promising. However, there have been no studies in patients with TR-MDD nor with the use of Facebook with the psychiatrist as “friend.” 60 TR-MDD patients were randomized to one of three groups: Facebook group with psychiatrist as “friend,” Facebook group without psychiatrist as “friend,” and control group (no Facebook use). Both Facebook groups spent at least 1 hour/day on Facebook, 7 days/week, during the 3 months. All patients maintained their usual pharmacotherapy. All participants were evaluated at baseline and at 1, 2, and 3 months for depressive symptoms using HAD17 and BDI-II. Results show that both Facebook groups had a decrease on HADM17 and BDI-II scores as well as higher remission and response rates than the control group, with better results if the psychiatrist was a “friend” on Facebook. Therefore, in TR-MDD, Facebook can be used as an effective enhancement therapy, adjuvant to pharmacological therapy with regular consultations, especially if the psychiatrist is the patient's online “friend.”

## 1. Introduction

Although several strategies have been used in an attempt to increase the successful outcome of treatment-resistant major depressive disorder (TR-MDD), remission rates remain low, and different alternative approaches have been suggested, with physical exercise showing promising results [1–5]. However, given the usual fatigue, tiredness, and reluctance to leave home of MDD patients, and most especially TR-MDD patients,   social networks such as Facebook can be particularly interesting, allowing them to freely communicate with others without having to leave the haven of their home. On the other hand, the possibility of overcoming the common social self-exclusion of these patients may contribute to reducing the typical anhedonia, thus initiating the desired remission process.

In recent years some studies have been conducted on the influence of the Internet and social networks on depressive symptoms and suicidal ideation. The implementation of cognitive behavioral therapy (CBT) interventions via the Internet has been shown to improve symptoms in adults with depression and has high adherence rates [6, 7], being more effective if there is contact with the therapist [8] and if it is personalized [9]. College students who use Facebook have fewer depressive symptoms [10], and those who explicitly express these symptoms and receive online support from their “friends” have higher self-esteem and sense of well-being [11]. CBT and social support via the Internet have also been shown to be effective in mitigating loneliness among the elderly [12] and in reducing suicidal ideation [13]. However, there have been no studies on patients with TR-MDD nor with the use of Facebook with the psychiatrist as “friend.”

## 2. Methods

### 2.1. Study Design

Prospective, randomized, open trial, three-arm, parallel assignment.

### 2.2. Participants

Between January 2011 and June 2012, 60 patients attending the outpatient psychiatry clinic at Clínica Médico-Psiquiátrica da Ordem, diagnosed with MDD according to DSM-IV criteria, taking combined therapy in doses considered adequate [14] during at least 9 months, and considered treatment-resistant given they failed to reach symptomatic remission after at least 2 adequate antidepressant trials [15], were included in the study. APA guidelines were followed regarding adequacy of dose and duration of treatment and class of medications administered [14]. All patients were medicated with nonsedating antidepressants in doses considered therapeutic [16]. Clomipramine, maprotiline, and amitriptyline were used as tricyclic antidepressants at a dose of 125–150 mg/day, as SSRIs fluoxetine, escitalopram, paroxetine, and sertraline were used, at doses of 20–40 mg/day, 20 mg/day, 20–40 mg/day, and 100–150 mg/day, respectively; venlafaxine was used as SNRI at a dose of 150 mg/day; agomelatine was used at a dose of 25–50 mg/day. When considered appropriate, lorazepam was used as anxiolytic at a dose of 1–2.5 mg/day. These 60 patients were randomized following a 1 : 1 : 1 scheme to one of three groups: control (*N* = 20), Facebook use (*N* = 20), and Facebook use with the psychiatrist as “friend” (*N* = 20). The protocol was approved by the Institutional Review Board of Clínica Médico-Psiquiátrica da Ordem. All participants provided written informed consent.

### 2.3. Inclusion and Exclusion Criteria

Inclusion criteria were as follows: (1) being males or females aged 18–70 years; (2) being able and willing to provide written informed consent; (3) being diagnosed for treatment-resistant major depressive disorder; (4) having ilimited Internet access; (5) not being prior users of Facebook.

Exclusion criteria were as follows: (1) psychiatric comorbidities; (2) relevant clinical comorbidities; (3) psychotic symptoms; (4) imminently suicidal; (5) undergoing psychotherapy; (6) change of pharmacological therapy less than 6 weeks prior to the beginning of the study.

### 2.4. Study Protocol

All patients maintained their usual pharmacotherapy throughout the study and were randomized to one of three groups: *Facebook group with psychiatrist as “friend” (Group FF)*: 20 patients that did not use Facebook before and started spending at least 1 hour per day on Facebook, 7 days per week, during the 3 months. During this Facebook time, they could interact with their own “friends” and could talk freely with the psychiatrist, who was one of the “friends” (the psychiatrist kept the presence green light on, so that patients in this group could talk to him if they wanted to). *Facebook group without psychiatrist as “friend” (Group F)*: 20 patients that did not use Facebook before and started spending at least 1 hour per day on Facebook, 7 days per week, during the 3 months, were included. During this Facebook time, they could interact with their own “friends” but could not talk with the psychiatrist (since the psychiatrist was not their “friend” patients in this group could not see the psychiatrist presence green light on and therefore could not talk to him). *Control group (Group C):* 20 patients that continued to be followed at regular consultations with the psychiatrist were included. All participants were evaluated at baseline (time 0: before starting the protocol) and at 1, 2, and 3 months for depressive symptoms.

### 2.5. Depression Assessment

Depression was assessed by the HAMD17 total score [17] and the Beck depression inventory (BDI-II) [18]. Response and remission rates were based on HAMD17, with response defined as a decrease from baseline to endpoint of ≥50% on the HAMD17 total score and remission defined as an endpoint HAMD17 total score ≤7.

### 2.6. Compliance Assessment and Definition

All participants included in both the Facebook groups were given a diary and asked to register daily how much time they spent on Facebook. Compliance to both Facebook interventions was based on these diaries. Compliance was defined as spending at least 1 hour per day on Facebook, 7 days per week, during the 3 months.

### 2.7. Statistical Analysis

This study used an intent-to-treat analysis with no missing values. Baseline clinical characteristics were analysed using an analysis of variance (ANOVA). In the case of significant differences, post hoc tests using the Sidak correction were performed. Differences between and within treatment groups in the change from baseline to endpoint (3 months) and data concerning the 4 time points (baseline, 1, 2, and 3 months) were analysed using an analysis of covariance (ANCOVA) with baseline values as covariates. In the case of significant differences, post hoc tests using the Sidak correction were performed. Differences in response and remission between groups were assessed using the Fisher exact test, and the Bonferroni correction was applied when necessary. Tests were considered significant at *α* = 0.05 significance level (two-sided).

## 3. Results

### 3.1. Study Population and Baseline Values

Of the 60 participants included in the study, 3 from the Facebook group with the psychiatrist as “friend” were excluded from analysis given they only attended the first appointment and therefore did not start the study. The remaining 57 patients all completed the study; thus the overall drop-out rate was 5%. There was a 100% adherence to both Facebook interventions. There was no difference on HAMD17 or BDI baseline values between groups: HAMD17, Group C  16.35 ± 4.96, Group F 19.10 ± 5.57, and Group FF 17.65 ± 4.32, *P* > 0.05; BDI, Group C 17.35 ± 5.02, Group F 20.50 ± 4.38, and Group FF 19.90 ± 4.51, *P* > 0.05. There was no difference in pharmacological agents used between the three groups: *P* = 0.374 and *P* = 0.968, for the antidepressants and the anxiolytic, respectively.

### 3.2. Changes of HAMD17 and BDI over the 3-Month Study Period

**Figure 1:**
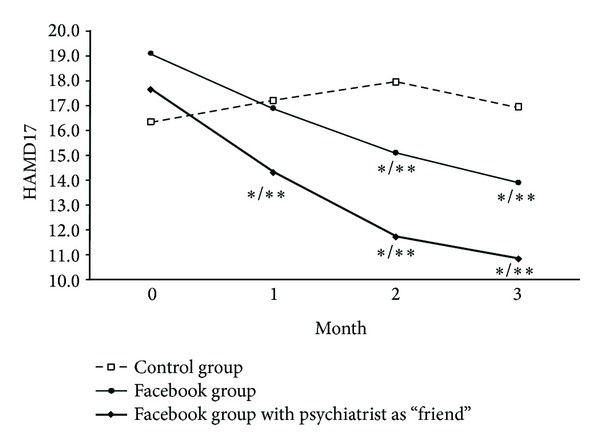
*P < 0.05 compared to baseline; **P < 0.05 compared to control group. *P* values from ANCOVA with baseline values as covariate.

**Figure 2:**
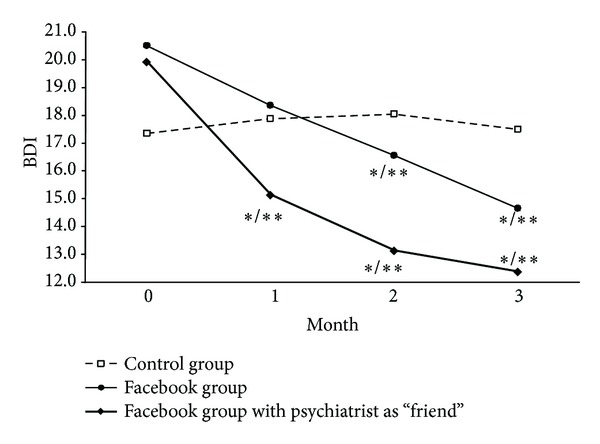
*P < 0.05 compared to baseline; **P < 0.05 compared to control group. *P* values from ANCOVA with baseline values as covariate.

Analysis of the 4 time points—baseline, 1, 2, and 3 months—shows that HAMD17 scores decreased significantly on both Facebook groups at months 2 and 3 compared to baseline, although Group FF showed a faster decrease, being significant after 1 month. The same pattern was observed when comparing both Facebook groups with the control group—Figure 1. The same pattern was observed concerning BDI-II scores—Figure 2.

### 3.3. Changes in Response to Treatment over the 3-Month Study Period

**Figure 3:**
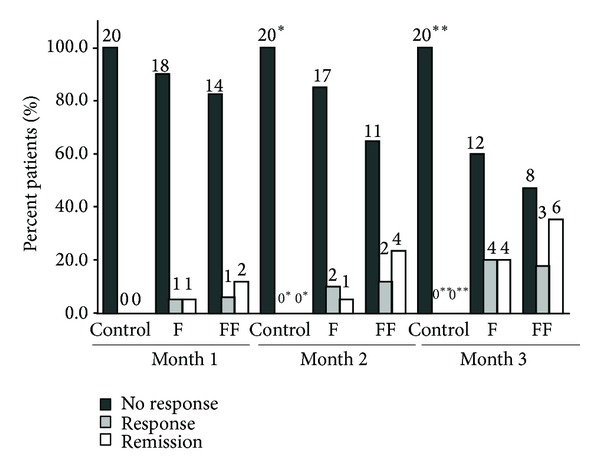
Group F: Facebook without psychiatrist as “friend”; Group FF: Facebook with psychiatrist as “friend.” *P < 0.05 compared to Group FF; **P < 0.05 compared to Group F and Group FF. *P* values from Fisher exact test. The Bonferroni correction was applied.

On month 2, there was a significant difference in the percentage of nonresponders, responders, and remitted patients between the control group and Group FF (nonresponders: Group C 100.0% and Group FF 64.7%; responders: Group C 0.0% and Group FF 11.8%; remitted patients: Group C 0.0% and Group FF 23.5%, *P* < 0.05). On month 3, this difference was statistically significant between the control group and the two Facebook groups (nonresponders: Group C 100.0%, Group F 60.0%, and Group FF 47.1%; responder: Group C 0.0%, Group F 20.0%, and Group FF 17.6%; remitted patients: Group C 0.0%, Group F 20.0%, and Group FF 35.3%, *P* < 0.05)—Figure 3.

## 4. Discussion

Since the invention of the press by Gutenberg in 1450, nothing has probably had such a strong impact on the way people interact as the Internet. However, the Internet represents not one revolution but several. The Internet itself and its most popular service, the World Wide Web, became commercially available in the early 1990s. Although it allowed people to access thousands of contents in their own computer, the so-called Information Highway was mainly a one-way street. In the late 1990s, the Web Logs, called Blogs, changed that, allowing the common individual to publish his own contents without the need of technical knowledge. The third revolution was the advent of Social Networks (SNS). Although there are several SNSs, Facebook became the standard SNS in the middle 2000s, thus effectively changing concepts like “friend,” “acquaintance,” or even “like.” Nowadays, we can in fact be connected to the whole world, learn what our friends are doing and saying, and answer back to them, even if we never met any of them in person.

The use of SNS by children and adolescents have raised several concerns, namely the occurrence of “Facebook depression,” defined as depression that develops when preteens and teens spend a great deal of time on social media sites and then begin to exhibit classic symptoms of depression [19]. Given this concern, several studies have been conducted to ascertain the potential risk of computer/Internet and SNS use on eliciting depression among adolescents, with apparently contradictory findings. Among high school students, online social networking was found to be related to depression [20], and among Korean adolescents a relationship was found between problematic Internet use, depressive symptoms, bipolar symptoms, and suicidal ideation [21]. However, none of these studies established a causal relationship. On the other hand, the Canadian Community Health Survey, which studied the association between hours of use of different media and health outcomes in youth aged 12–19 years, did not find an association between depression and computer/Internet use, concluding that media use is not universally harmful [22]. These results were corroborated by a study in older adolescent university students, which found no association between the use of social networking sites and either any depression or moderate to severe depression, concluding that counseling patients or parents regarding the risk of “Facebook depression” may be premature [23]. Moreover, studies among college students suggest that those who display depression symptoms on Facebook receive support and reinforcement from their online friends [11, 24], thus being more likely to discuss their depressive symptoms, which can be the first step for seeking help. Also, adolescents who receive positive feedback on their social networks profiles reported enhanced self-esteem and sense of well-being [25]. These results may be a potential explanation for the findings of a study including Turkish college students that showed that severe depression, among other variables, positively predicted Facebook addiction [26], and they could also be the causal relationship missing from the studies by Pantic et al. [20] and Park et al. [21]: depressed, bipolar, or potentially suicidal teenagers spend more time on Facebook because it helps them overcome at least some of the symptoms. This hypothesis is supported by a prospective study in adolescents and young adults that concluded that excessive Internet and video gaming use may be a symptom of mental health problems rather than a cause, and moderate Internet use is supportive of a healthy development [27].

Taken together, these results suggest that Internet and SNS use may have more positive than negative effects, and if this is true for adolescents, who are more prone to peer pressure, then it will be true for adults, who may even benefit more from the social interaction provided by SNS. Given the potential of reaching thousands at literally no costs while investing very little time, several studies on Internet-based cognitive behavioral therapy (iCBT) or psychodynamic psychotherapy (iPDT) programs started to emerge, many of them focusing on depressed populations or symptoms of depression and anxiety [6, 8, 9, 12, 28–34], with mostly positive results, albeit not in all cases. One study even delivered fully automated iCBT to a large healthy, nonpsychiatric adult population, with good results on increasing the mental well-being and self-rated scores of depression and anxiety of participants [35], and another concluded that these iCBT programs help reduce suicidal ideation in depressed patients, even though they are not specifically designed with this aim [13]. However, no studies have yet tried to take advantage of Facebook as a possible therapeutic tool in TR-MDD patients.

We designed two very simple, easy to deliver Facebook interventions in an attempt to increase response and remission rates of TR-MDD patients. These patients continued to take their usual pharmacotherapy and to go to regular consultations with the psychiatrist but were asked to start spending at least 1 hour per day, 7 days per week, on Facebook. One of the groups had the psychiatrist as “friend” while the other did not. No attempt was made to deliver any kind of therapy via Facebook. The control group continued with treatment as usual, taking their usual pharmacotherapy and going to regular consultations with the psychiatrist.

The overall drop-out rate was 5%, with 3 patients from the Facebook group with the psychiatrist as “friend” being excluded from the study given they only attended the first appointment and therefore did not start the study. Of the patients remaining in the study, there was a 100% adherence to both Facebook interventions. Different adherence rates of iCBT or iPDT programs have been reported, ranging from as low as 33.1% to as elevated as 100% [6, 9, 29, 31–33, 35]. This large discrepancy may be due to heterogeneity regarding severity of depression, existence of several comorbidities, recruitment strategy and design of the iCBT, and iPDT programs. Two recent studies did not find a relationship between symptom severity and adherence, but they were both in subclinical populations [35, 36]. A recent systematic review of studies in clinical populations concluded that less severe depression was a predictor for increased adherence [37], and a meta-analysis on iCBT programs for symptoms of depression and anxiety showed that the dropout rate is considerably higher in interventions without therapist support compared to interventions with therapist support [28]. This is not in accordance with our adherence rate of 100%, given all our patients were TR-MDD, and therefore particularly severe patients, and the adherence rates for both Facebook interventions were the same. We may speculate that this may be due to our study design, which did not include any structured iCBT or iPDT intervention, with the potential for dropout due to lack of motivation, lack of face-to-face contact, preference for taking medication, perceived lack of treatment effectiveness, or burden of the program [37]. All our patients continued taking their medication and attending regular consultations with the psychiatrist, and Facebook was not suggested as a means of treatment but simply as a means of distraction, making new friends, reconnecting to lost friends, and talking online.

Regarding HAMD17 and BDI-II scores, both Facebook groups showed improvements compared both to baseline and to the control group, and also, at the end of the study, more patients had remitted on both Facebook groups compared to the control group. However, the group that had the psychiatrist as “friend” improved faster and at the end of the study more patients in this group had remitted. Although it is not possible to directly compare our results with the reported ones, given our study design was unique, we may establish some parallels with other interventions. iCBT, iPDT, and problem-solving therapy interventions with therapist support have been reported to be associated with larger effect sizes in reducing depressive and anxiety symptoms than interventions without therapist support [28, 30, 33] or less support [31], and a recent review provides evidence for a strong correlation between the degree of support and outcome in iCBT [8], with support provided by email, telephone, or chat websites. On the other hand, a study on patients with MDD or dysthymia suggested that Internet-delivered treatments for depression can be effective whether support is added or not [29], and one study in a subclinical population corroborated these results [32]. Providing Internet access to older adults showed a trend towards decreased loneliness and depression, but with no statistical significance [34], and a recent meta-analysis showed that this type of training interventions in older adults is significantly effective in decreasing loneliness but ineffective in decreasing depression. Again, study participants showed low levels of depression at baseline and therefore there was little room for improvement [12]. It is our opinion that our study design may be considered a combination of some form of iCBT or iPDT with encouragement of Internet use, with the group having the psychiatrist as “friend” being regarded as the most supported group, since the simple fact of using Facebook and interacting with online friends provides support. This support may be regarded as a form of unstructured iCBT or iPDT, since patients will talk to their online friends about their feelings and these will try to help them with words of encouragement and positive reinforcement. Also, if patients are using Facebook it is natural that they will increase their Internet use overall, which may distract them from their own problems, for instance, by accessing websites on subjects that interest them. Of notice is the fact that only 3 patients in the group that had the psychiatrist as “friend” contacted the psychiatrist during the 3-month study period, and all these contacts were questions regarding medication, and therefore there was no actual support from the psychiatrist. We can only speculate that simply knowing the psychiatrist was there for them if they needed support (the psychiatrist kept the presence green light on, so that patients in this group could talk to him if they wanted to) gave them some sense of comfort and assurance, being enough for them to improve faster and show higher levels of remission rates. Therefore, our results are in accordance with previous studies showing positive outcomes in depressed populations with Internet-based interventions, with the most supported group showing faster results and increased remission rates. The fact that these results are different from some reports on subclinical populations [12, 32, 34] and even one study on patients with MDD or dysthymia [29] may reflect the severity of depression. Our patients are particularly severe patients, with TR-MDD, which are willing to try anything that may potentially help them to get better, and therefore will be extremely motivated to enroll and adhere to such a simple intervention that requires very little effort. The simplicity of the study design, the fact that it is not demanding, and the patients' motivation certainly influenced the observed positive outcomes.

Finally, since the only 3 contacts with the psychiatrist were doubts concerning medication, this Facebook approach with the psychiatrist as “friend” may also contribute to the enhancement of pharmacotherapy adherence rates, which are known to be poor in these patients.

As every study, this study had some limitations. There was no assessment of compliance to medication, although there is no indication that patients did not comply. Another limitation was that the study was not blinded, with the possible inherent bias associated to open trial studies. Also, this kind of intervention is only possible if patients have unlimited Internet access at home, since patients accessed Facebook from home.

## 5. Conclusions

At a time of global economic and social crisis, due to which depression is increasing, with scarce clinical resources and resistance rates to antidepressants still considerable, Facebook can be used as an effective enhancement therapy, adjuvant to pharmacological therapy with regular consultations, in TR-MDD. Our results show that this kind of intervention had an adherence rate of 100%, is beneficial to the patient both in terms of improvement in depressive symptoms and in remission rates, and has no associated costs, being more effective if the psychiatrist is the patient's “friend” on Facebook. According to our results, psychiatrists can be the patient's “friends” on Facebook, given there was no misuse or abuse of this “friend” relationship: during the 3 months of the study, only 3 patients contacted the psychiatrist with doubts concerning medication. This type of intervention is an attractive, effective, and inexpensive option as an adjuvant therapy for TR-MDD patients, for whom even polypharmacotherapy has shown poor results.

## References

[1] Mota-Pereira J, Silverio J, Carvalho S, Ramos J, Ribeiro JC (2012). Physical exercise and major depressive disorder—where do we stand?. *Current Psychopharmacology*.

[2] Mota-Pereira J, Carvalho S, Silverio J (2011). Moderate physical exercise and quality of life in patients with treatment-resistant major depressive disorder. *Journal of Psychiatric Research*.

[3] Mota-Pereira J, Ribeiro JC, Fonte D, Carvalho S, Ramos J, Silverio J, Simmons JA, Brown AC (2013). Exercise intervention in treatment-resistant Major Depressive Disorder: benefits of accelerometer monitoring. *Aerobic Exercise: Health Benefits, Types and Common Misconceptions*.

[4] Mota-Pereira J, Silverio J, Carvalho S, Ribeiro JC, Fonte D, Ramos J (2011). Moderate exercise improves depression parameters in treatment-resistant patients with major depressive disorder. *Journal of Psychiatric Research*.

[5] Mota-Pereira J, Silverio J, Fonte D, Carvalho S, Ramos J, Ribeiro JC, Simmons JA, Brown AC (2013). Moderate exercise as an adjuvant therapy for treatment-resistant Major Depressive Disorder: 6 month follow-up. *Aerobic Exercise: Health Benefits, Types and Common Misconceptions*.

[6] Dear BF, Zou J, Titov N, Lorian C, Johnston L, Spence J (2013). Internet-delivered cognitive behavioural therapy for depression: a feasibility open trial for older adults. *The Australian and New Zealand Journal of Psychiatry*.

[7] Crabb RM, Cavanagh K, Proudfoot J, Learmonth D, Rafie S, Weingardt KR (2012). Is computerized cognitive-behavioural therapy a treatment option for depression in late-life? A systematic review. *The British Journal of Clinical Psychology*.

[8] Johansson R, Andersson G (2012). Internet-based psychological treatments for depression. *Expert Review of Neurotherapeutics*.

[9] Johansson R, Sjoberg E, Sjogren M, Johnsson E, Carlbring P, Andersson T (2012). Tailored vs. standardized internet-based cognitive behavior therapy for depression and comorbid symptoms: a randomized controlled trial. *PloS ONE*.

[10] Wright KB, Rosenberg J, Egbert N, Ploeger NA, Bernard DR, King S (2013). Communication competence, social support, and depression among college students: a model of facebook and face-to-face support network influence. *Journal of Health Communication*.

[11] Moreno MA, Jelenchick LA, Egan KG (2011). Feeling bad on facebook: depression disclosures by college students on a social networking site. *Depression and Anxiety*.

[12] Choi M, Kong S, Jung D (2012). Computer and internet interventions for loneliness and depression in older adults: a meta-analysis. *Healthcare Informatics Research*.

[13] Watts S, Newby JM, Mewton L, Andrews G (2012). A clinical audit of changes in suicide ideas with internet treatment for depression. *BMJ Open*.

[14] American Psychiatric Association (2010). *Practice Guideline for the Treatment of Patients with Major Depressive Disorder*.

[15] Malhi GS, Parker GB, Crawford J, Wilhelm K, Mitchell PB (2005). Treatment-resistant depression: resistant to definition?. *Acta Psychiatrica Scandinavica*.

[16] Taylor D, Paton C, Kapur S (2009). *The Maudsley Prescribing Guidelines*.

[17] Hamilton M (1960). A rating scale for depression. *Journal of Neurology, Neurosurgery, and Psychiatry*.

[18] Kessler RC, Abelson J, Demler O (2004). Clinical calibration of DSM-IV diagnoses in the World Mental Health (WMH) version of the World Health Organization (WHO) Composite International Diagnostic Interview (WMH-CIDI). *International Journal of Methods in Psychiatric Research*.

[19] O’Keeffe GS, Clarke-Pearson K, Mulligan DA (2011). Clinical report—the impact of social media on children, adolescents, and families. *Pediatrics*.

[20] Pantic I, Damjanovic A, Todorovic J, Topalovic D, Bojovic-Jovic D, Ristic S (2012). Association between online social networking and depression in high school students: behavioral physiology viewpoint. *Psychiatria Danubina*.

[21] Park S, Hong KE, Park EJ, Ha KS, Yoo HJ (2013). The association between problematic internet use and depression, suicidal ideation and bipolar disorder symptoms in Korean adolescents. *The Australian and New Zealand Journal of Psychiatry*.

[22] Casiano H, Kinley DJ, Katz LY, Chartier MJ, Sareen J (2012). Media use and health outcomes in adolescents: findings from a nationally representative survey. *Journal of the Canadian Academy of Child and Adolescent Psychiatry*.

[23] Jelenchick LA, Eickhoff JC, Moreno MA (2013). Facebook depression?. *Social Networking Site Use and Depression in Older Adolescents*.

[24] Moreno MA, Christakis DA, Egan KG, Jelenchick LA, Cox E, Young H (2012). A pilot evaluation of associations between displayed depression references on Facebook and self-reported depression using a clinical scale. *The Journal of Behavioral Health Services & Research*.

[25] Valkenburg PM, Peter J, Schouten AP (2006). Friend networking sites and their relationship to adolescents’ well-being and social self-esteem. *Cyberpsychology and Behavior*.

[26] Koc M, Gulyagci S (2013). Facebook addiction among Turkish college students: the role of psychological health, demographic, and usage characteristics. *Cyberpsychology, Behavior and Social Networking*.

[27] Romer D, Bagdasarov Z, More E (2013). Older versus newer media and the well-being of United States youth: results from a national longitudinal panel. *The Journal of Adolescent Health*.

[28] Spek V, Cuijpers P, NykIíček I, Riper H, Keyzer J, Pop V (2007). Internet-based cognitive behaviour therapy for symptoms of depression and anxiety: a meta-analysis. *Psychological Medicine*.

[29] Berger T, Hämmerli K, Gubser N, Andersson G, Caspar F (2011). Internet-based treatment of depression: a randomized controlled trial comparing guided with unguided self-help. *Cognitive Behaviour Therapy*.

[30] de Graaf LE, Hollon SD, Huibers MJH (2010). Predicting outcome in computerized cognitive behavioral therapy for depression in primary care: a randomized trial. *Journal of Consulting and Clinical Psychology*.

[31] Johansson R, Ekbladh S, Hebert A, Lindstrom M, Moller S, Petitt E (2012). Psychodynamic guided self-help for adult depression through the internet: a randomised controlled trial. *PloS ONE*.

[32] Morgan AJ, Jorm AF, Mackinnon AJ (2013). Self-help for depression via e-mail: a randomised controlled trial of effects on depression and self-help behaviour. *PloS ONE*.

[33] Van Straten A, Cuijpers P, Smits N (2008). Effectiveness of a web-based self-help intervention for symptoms of depression, anxiety, and stress: randomized controlled trial. *Journal of Medical Internet Research*.

[34] White H, McConnell E, Clipp E (2002). A randomized controlled trial of the psychosocial impact of providing internet training and access to older adults. *Aging and Mental Health*.

[35] Powell J, Hamborg T, Stallard N, Burls A, McSorley J, Bennett K (2013). Effectiveness of a web-based cognitive-behavioral tool to improve mental well-being in the general population: randomized controlled trial. *Journal of Medical Internet Research*.

[36] Wojtowicz M, Day V, McGrath PJ (2013). Predictors of participant retention in a guided online self-help program for university students: prospective cohort study. *Journal of Medical Internet Research*.

[37] Christensen H, Griffiths KM, Farrer L (2009). Adherence in internet interventions for anxiety and depression. *Journal of Medical Internet Research*.

